# Cooper on Dislocations and Fractures of the Joints

**Published:** 1832

**Authors:** 


Art. V.—*A Treatise on Dislocations and Fractures of the Joints.* By Sir Astley Cooper, Bart. F. R. S., Sergeant Surgeon to the King, &c. &c. To which are added the Notes and References to the first American Edition. By John D. Godman, M. D., &c.

It must always be with peculiar pleasure that the student or practitioner of medicine sits down to the perusal of a work like this of Sir Astley Cooper. An individual of great and practical mind, with opportunities for observation unequalled, with an ambition of a high order, yet chastened down to its appropriate sphere of honorable professional ambition, brings all the learning of the past, and all the experience of the present age, to assist him in constructing a treatise upon one of the most important departments of Surgery, which will leave nothing for future historians to complete, either in the description of the possible varieties of disease, or in the invention of remedial measures. Such a work we consider the *Treatise on the Dislocations and Fractures of the Joints.*

The first chapter is of course devoted to *Dislocations in general.* In enumerating the various sources of difficulty which prevent the surgeon from forming a satisfactory diagnosis in cases of dislocation, Sir Astley lays particular stress upon the importance of a knowledge of the anatomy of the Joints, and comments with great justice upon the almost universal practice among students after studying with great care and accuracy the arrangement of the muscular, vascular, and nervous systems, to pass by the structure of the joints as an affair of little moment. From this carelessness originate frequent mistakes; for the dislocations of the elbow, the shoulder, or the hip, are frequently not to be detected without accurate anatomical knowledge. This neglect is the more inexcusable, inasmuch as the principal part of surgical practice consists in injuries of this nature. It is therefore of the utmost importance "that the form of the extremities of the bones, their mode of articulation, the ligaments by which they are connected, and the direction in which their most powerful muscles act, should be well understood."

After dislocation, the limb generally assumes a determinate position—in consequence of the action of the muscles—and after a short interval, it becomes so fixed in its position by the tonic contraction of the muscles, that it is moved with great difficulty.

The length of the limb is altered, and it is generally shortened; sometimes, however, it is lengthened, and then the muscles, being put very much upon the stretch, are lacerated, and an effusion of blood, in proportion to the extent of the injury, takes place, increasing very much the difficulty of diagnosis.

The pressure of the head of the bone frequently produces very troublesome effects—from pressing upon a large nerve, for instance, paralysis or severe pain may result; and a dislocated clavicle has been known to endanger life by obstructing the œsophagus.

A sensation of crepitus is sometimes experienced after dislocation, from the effusion of adhesive matter into the joint and bursæ—"a circumstance of which every practitioner ought to be aware." The very fact of the practitioner being aware of this circumstance seems occasionally to be attended with its inconveniences, as if it were necessary that ignorance should have something to keep it in countenance. It happened once with ourselves to be very much mortified by having a distinguished surgeon pronounce, that the crepitus by which we recognized a fracture of the fibula near the knee, was merely the rubbing of the bursæ. The subsidence of the swelling, however, and the inability of the old patient to use the limb for two years, convinced both patient and physician of the existence of fracture.

Dissection shows the capsular ligament to be lacerated, to a very considerable extent, and the peculiar ligaments of the joint to be torn through. The tendon of the biceps which serves the double purpose of ligament and tendon, is not lacerated when the arm is dislocated into the axilla, the tendon of the subscapularis, however, is, and upon the extent of the laceration very much depends the facility of reduction.

The dissection of old dislocations frequently shows so wonderful a power of accommodation to the necessities of the case, that we shall extract Sir Astley's remarks upon the subject.

"*Dissections of old Dislocations, &c.*—The appearance of joints which have long been dislocated, depends not only on the length of time that has elapsed from the accident, but also on the structure upon which the head of the dislocated bone is thrown; for, if it be found embedded in muscle, its articular cartilage remains, and a new capsular ligament forms around it, which does not adhere to its cartilaginous surface. This ligament, in dislocations of the femur, contains within it the head of the bone, with the lacerated portion of the ligamentum teres united to it. In these instances, the bones themselves undergo little change. The capsular ligament is formed from the surrounding cellular tissue; which being pressed upon by the head of the bone, becomes inflamed, thickened, and condensed. By this means a substance is produced somewhat less dense than original ligament, but still possessing sufficient firmness to bear considerable pressure, and furnish some degree of support.

*Head of the bone resting on another bone, &c.*—But if the head of the dislocated bone be placed on the surface of another bone, or upon a thin muscle over it, that muscle becomes absorbed, and the bone undergoes a remarkable change; thus it is found, if the dislocation be not reduced, that both the ball and the bone that receives it, are changed in their form. The pressure of the head of the bone produces absorption of the periosteum, and of the articular cartilaginous surface of the head of the bone; a smooth hollow surface is formed, and the ball becomes altered in its shape to adapt it to its new surface; and whilst this absorption proceeds upon the part on which the head of the bone rests, an ossific deposite takes place around it from the periosteum, which is there irritated, but not absorbed. By the deposition of this bony matter between the periosteum and the original bone, a deep cup is formed to receive the head of the bone, and perhaps no instances can be adduced which more strongly mark the powers of nature in changing the form of parts to accommodate them to new circumstances, than these effects of dislocation.

The new cup which is thus formed, sometimes so completely surrounds the neck of the bone, as to prevent its being separated without fracture, and the socket is smoothed upon its internal surface, so as to have no projecting parts, which can interrupt the motion of the bone in its new situation.

The muscles losing their action, become diminished in length, in proportion to the displacement of the bone towards their origin; and if the dislocation has been long unreduced, they lose their flexibility, and tear rather than yield to extension."

Dislocations, although generally produced by violence, sometimes result from other causes—from *relaxation of the ligaments;* this occurs most frequently in the shoulder, in the patella, and in rare cases in the hip. Individuals are frequently met with who can dislocate the shoulder at pleasure.

Dislocations may arise from paralysis; especially in those joints in which the structure of the articulation is principally preserved by the action of the muscles. They are produced also by ulceration.

Fracture and dislocation frequently result from the same injury, and indeed such is the structure of some of the joints, that the latter can scarcely take place without the former, as in the ancle joint, for instance. When these two accidents are combined, it is generally necessary, after firmly securing the limb by splints, to reduce the dislocation without loss of time, it cannot afterward be done without endangering the union of the bone.

Compound dislocations are attended with great constitutional irritation and consequent danger to life.

"When a joint is opened, inflammation of the lacerated ligaments and synovial surface speedily succeeds; in a few hours suppuration begins, and granulations arise from the surface of the secreting membrane; which, being of the mucous kind, is more disposed to the suppurative, than to the adhesive inflammation. But the same process does not immediately ensue upon the extremity of the bone, because it is covered by the articular cartilage. This cartilage, before the cavity fills with granulations, becomes absorbed, by an ulcerative process instituted on the ends of the bones, but sometimes beginning from the synovial surface. The bone inflames, the cartilage becomes ulcerated, numerous abscesses are formed in different parts of the joint, and at length granulations spring from the extremities of the bones deprived of their cartilages, and fill up the cavity; generally these granulations become ossified, and anchylosis succeeds; but sometimes they remain of a softer texture, and some degree of motion in the joint is gradually regained.

This process of filling up joints requires great general as well as local efforts; a high degree of irritation is produced; and if the constitution be weak, the patient, to preserve his life, is obliged to submit to amputation.

*Injury to muscles, blood-vessels, etc.*—In addition to the above circumstances, the violence necessarily inflicted on the parts in compound dislocations, the injury of the muscles and tendons, and the laceration of blood-vessels, necessarily lead to more important and dangerous consequences than those which follow simple dislocations."

Sir Astley Cooper has seen two instances of this accident at the shoulder, and one at the knee—at the elbow, ancle, and wrist it frequently occurs.

It is not necessary perhaps to mention, that the muscles constitute so great a preservative against dislocation, that it generally takes place when they are unprepared to resist; or, in consequence of their sudden and convulsive action when the limb is in an untoward position.

Dislocations are rare in old persons—the bones from their brittleness rather giving way. They are also comparatively rare in the young—the epiphyses generally yielding.

*Obstacles to the Reduction of a Dislocated Limb.* These may exist in the structure of the joint; such as arises from the lip of the bone constituting the socket of the thigh, or from the head of the bone being much larger than the cervix, as is the case with the radius.

A more serious source of difficulty arises from the ligaments which surround the joint. The capsular ligaments from their delicacy, can have but little tendency either to prevent a dislocation, or to resist its reduction. The joints, however, are provided with ligaments accommodated to the nature and extent of their motions, and the injuries to which they are exposed, and their structure is still further strengthened by the tendons of the muscles being inserted in such manner as to serve the purpose of ligaments. These ligaments must of necessity generally be lacerated before dislocation can take place. They do, however, occasionally escape laceration, at the time the bone slips from its socket, and afterward, present serious difficulties in the way of its reduction.

The contraction of the muscles offers the principal obstacle to reduction. As soon as dislocation takes place, the muscles contract, to accommodate themselves to the new position of the bone, and this contraction continues until the structure of the muscle becomes permanently altered, by a deposition of dense fibrous matter. When force is applied to overcome this contraction, the muscles oppose a resistance proportioned to the length of time since the accident, and this resistance after a time becomes insurmountable.

In old dislocations, other difficulties occur. Adhesions form between the extremity of the bone and the surrounding parts—the socket becomes filled with adhesive matter, so that the bone, if reduced, would not remain in its original situation—and lastly, a new bony socket is sometimes formed, completely confining the head of the bone.

The means to be employed for the reduction of dislocations, are both constitutional and mechanical. Force alone is insufficient to overcome resistance of the muscles, since the degree of it necessary for the reduction of many dislocations would be liable to produce violence and injury. The constitutional means to be employed are those which cause relaxation of the muscles by producing a disposition to syncope—bleeding, the warm bath, and nausea. If bleeding is resorted to, it is scarcely necessary to inform the student, that it should be taken under such circumstances as will secure its full influence with as little expense to the system as possible. The blood should be taken, therefore, from a large orifice, while the patient is standing. Dr. Gibson thinks that a knowledge on the part of the patient of the intention of the surgeon to make him faint, makes the abstraction of a much greater quantity of blood necessary before the object is secured.

The *warm bath* is resorted to where circumstances connected with the general health of the patient render bleeding inadmissible. The temperature should be from 100 to 110, and, as the object is to relax the muscular system, should be continued until some degree of faintness is produced.

*Nauseating doses of tartar emetic* are frequently used for the purposes of diminishing the power of the muscles, but as this medicine is uncertain in its operation—frequently producing vomiting, which is both unnecessary and inconvenient, Sir Astley's custom is merely to use it in such a manner as to keep up the relaxation produced by the other remedies.

*Opium*, Sir Astley suggests, in large doses, might be useful. The relaxing effects of *tobacco* and of *intoxicativn by ardent spirits* have been successfully resorted to, but the practice is not likely to be generally followed.

In resorting to the necessary mechanical expedients, it is of the greatest consequence to have the bone in which the socket of the joint is placed, firmly fixed before extension is commenced; and the greater attention to this point is necessary in proportion to the mobility of this bone. One of the principal sources of difficulty in the reduction of a dislocated os humeri, arises from the mobility of the scapula. The joint being properly secured for the purpose of counter-extension, the extending power is to be applied gradually, so as rather to overcome the resistance of the muscles by fatigue than to overpower them by violence.

*The Compound Pully* for this purpose, where considerable force is necessary, has a great superiority over the action of assistants applied directly to the limb. Its effect may be gentle, long-continued, and completely under the control of the surgeon. The action of assistants, on the other hand, is necessarily irregular, sudden, and often ill-directed, and as their muscles are as liable to become fatigued as those of the patient, there is danger of their relaxing their efforts, perhaps at the very moment when the resistance is about to give way. Ambrose Parey was the first to use the pully. Most writers on Surgery since his time have adverted to them, but they have not duly appreciated their importance.

*A relaxed condition of the stronger muscles*, by a judicious position of the limb, is frequently of great importance, and in difficult cases, should always be consulted as far as practicable.

*Should the extension be applied to the dislocated bone, or on the limb below?* Mr. Boyer prefers the latter mode; Sir Astley, except in the case of a dislocation of the shoulder, prefers the former.

*The influence of volition* upon the muscles may be frequently turned to very good account, by causing the patient to make some muscular exertion, while the extension is kept up, the opposition of the muscles often ceases, and the bone slips into its place in a moment.

A great deal has been said about the length of time at which a dislocation may be reduced, and some surgeons in this country have acquired great eclat by successful attempts at reduction many months after the accident. As these accounts have been republished by most of the surgical writers in this country, in a manner calculated to excite sanguine expectations that are not likely to be very generally realised, it may not be improper to give the opinion of Sir Astley Cooper upon this subject.

"*Old dislocations not to be reduced.*—I believe that much mischief is produced by attempts to reduce dislocations of long duration in very muscular persons. I have seen great contusion of the integuments, laceration and bruises of muscles, tension of nerves, inducing an insensibility and paralysis of the hand, occasioned by an abortive attempt to reduce dislocation of the shoulder; so that the patient's condition has been rendered much worse than before. In such cases, even when the bone is replaced, it has often proved rather an evil than a benefit, from the violence of the extension.

In those instances in which the bone remains in the axilla, in dislocations of the shoulder, a serviceable limb and very extensive motions of it may be regained, although reduction has not been effected. Captain S——, who had dislocated his shoulder four years before, called to show me how much motion he had recovered, although the arm still remained unreduced.

*Time for attempting reduction.*—I am of opinion, that three months after the accident for the shoulder, and eight weeks for the hip, may be fixed as the period at which it would be imprudent to make the attempt at reduction, except in persons of extremely relaxed fibre, or of advanced age. At the same time, I am fully aware, that the shoulder has been reduced at a more distant period than that which I have mentioned; but, in most instances, the reduction has been attended with the results I have just been deprecating.

In cases of unreduced dislocation, the only course which the surgeon can adopt, after the inflammation which the injury produces has subsided, is, to advise motion of the limb, and friction of the injured part:—The former to produce a new cavity for the head of the bone, to assist in forming a new ligament, and to restore action to muscles, which they would otherwise lose by repose;—the latter, to produce absorption, and remove the swelling and adhesions which the accident has caused."

These views are confirmed by the bad success of several late attempts at reduction in this country, which have been recorded, and probably of many more which have not been recorded.

*Particular Dislocations.—Dislocations of the hip-joint* come first under the author's notice. Upon this subject he is very full and explicit, illustrating every possible variety of the accident, with its symptoms, diagnosis, consequences, &c., by numerous cases, but our limits do not permit us, at present, to examine this subject. To this is appended an account of the *fractures of the upper part of the thigh.*

This subject is one of very great importance, as well on account of the frequent occurrence of accidents of this kind, as from the difficulty which frequently exists of forming a diagnosis between fracture and dislocation.

These accidents, Sir Astley states, are much more numerous than dislocations; for while there are not received into the hospitals of Guy's and St. Thomas's (containing about nine hundred patients) more, upon an average, than two such dislocations in a year, the wards are seldom without an example of fracture of the upper part of the thigh-bone.

The result of the fracture will differ very much, according to its situation—a fact that is particularly deserving of the attention of the young surgeon. The varieties of the fracture may be classified in the following manner:—1. Those which occur through the neck of the bone, within the capsular ligament.—2, Through the neck of the bone, at its junction with the trochanter.—3. Through the trochanter.

Fractures within the capsular ligament, according to Sir Astley, are never united by osseous union. A great difference of opinion has prevailed among surgeons respecting the possibility of uniting fractures of the cervix femoris. Sir Astley has attempted to reconcile the discrepancy, by proving that fractures within the ligament never unite, while those occurring without, heal, as under other circumstances. To this point he adduces a great body of evidence, and extends the principle to fractures of the patella, the olecranon, the condyles of the humerus, and of the coronoid process of the ulna—viz. that they unite only by ligament.

The fracture within the ligament occurs most frequently in women, on account of a peculiarity in their structure, which will be readily understood by every student of anatomy who has paid any attention to this subject.

It occurs also in those advanced in life. Out of between three and four hundred cases of fracture of the upper part of the thigh-bone, which Sir Astley has seen, but two cases of fracture within the ligament have occurred in persons under fifty years of age, aud one of these was in a patient aged thirty-eight, who had an aneurism of the iliac artery. After this age dislocations seldom take place except in persons whose constitution is very robust.

The principles upon which this diversity is to be explained, are to be sought for,—first, In the general changes which the bones undergo, with the advance of age, by which their structure becomes lighter, softer, and more brittle;—secondly, In the peculiar change which takes place in the neck of the thigh-bone.

"Even the neck of the thigh-bone in aged persons is sometimes undergoing an interstitial absorption, by which it becomes shortened, altered in its angle with the shaft of the bone, and so changed in its form as to give an idea, upon a superficial view, that it has been the subject of fracture, thus leading persons into the erroneous supposition, that the bone has been partially broken and reunited; but it requires very little knowledge of anatomy to distinguish in the skeleton, the bone of advanced age from that of the middle period of life.

The neck of the thigh-bone in adult persons of middle age, has a close cancellated structure, with considerable thickness of the shell which covers it; but in old subjects, the cancellated structure of the shaft of the bone, which is formed of a course net-work, loaded with adipose matter, is often extended into the neck of the bone, and the shell which covers it becomes so thin, that when a section is made through the middle of the head and cervix, it is found diaphanous; of this I have several specimens. As the shell becomes thin, ossific matter is deposited on the upper side of the cervix, opposite the edge of the acetabulum, and often a similar portion at its lower part, and thus the strength of the bone is in some degree preserved; this state may be frequently seen in old persons. Mr. Steel, of Berkhampstead, one of the most intelligent surgeons, and most respectable men I know, gave me the thigh-bone of a person thus altered, whose age was ninety-three.

When the absorption of the neck proceeds faster than the deposit on its surface, the bone breaks from the slightest causes, and this deposit wears so much the appearance of an united fracture, that it might be easily mistaken for it. Before the bone thus alters, we sometimes meet with a remarkable buttress shooting up from the shaft of the bone into its head, giving it additional support to that which it receives from the deposit of bone upon its external surface. But another change is also produced from disease, of which the following is the history, and which directly applies to the subject before us.

Old bed-ridden and fat persons, (generally females), are often brought into our dissecting-room with some of their bones broken (and more frequently the thigh-bone than any other) in being removed from the grave. If the cervix femoris of such persons be examined, it will be found that the head of the bone is sunken down upon its shaft, and that the neck of the thigh-bone is shortened, so that its head is in contact with the shaft of the bone opposite to the trochanter minor; and at the part at which the ligament is inserted into the neck of the bone, the phosphate of lime is absorbed, and a ligamento-cartilaginous substance occupies its place; either extending entirely through the neck of the bone, or partially, so that one section exhibits signs of it, and in another it is wanting. The bone, in some cases, is so soft and fragile, both in its trochanter and head, that it will scarcely bear the slightest handling; and the motion of the thigh-bones in the acetabulum is almost entirely lost, so that the persons must have had little use in their lower extremities.

This explanation will also account for the slight causes, by which this fracture is produced—a mis-step, or some other trifling accident.

The causes which Sir Astley assigns for the want of ossific union, are, 1. *The want of proper apposition.*—He thinks himself authorized, from much observation and from numerous experiments, to lay it down as a principle, that perfect union never takes place when the extremities of the broken bone are not in contact. When the broken extremities of a bone are separated entirely, either by the action of the muscles, or other causes, the deposit of osseous matter, which forms the callus, is very imperfect, and if they are partially in contact the deposit is very abundant at the point of union, and less so at the other points. Now the tendency of the muscles in a fracture of this kind, is to keep the bones separated, in a manner, that it is impossible to prevent; and this tendency, when the capsular ligament is not torn, is still further promoted by the increased secretion of the synovia, which arises from the inflammation that now takes place, by which the capsule is so distended as to keep the ends of the bone asunder, until the inflammatory process has ceased, and the disposition to heal has subsided.

The principal cause, he thinks, 2. *is the want of action in the head of the bone.* The head of the bone is principally supplied by the vessels of the periosteum of the cervix. If, therefore, as is generally the case, the periosteum be torn when the fracture takes place, the circulation is necessarily very much interrupted, consequently no deposit of bone or cartilage takes place, but the deposit which does take place consists of ligamentous matter, covering the surface of the cancellated structure, with little patches, like ivory, on the head of the bone. The following description of the appearances on dissection, seem to confirm the author's views.

"*Dissection of this fracture.*—The appearances which are found on the dissection of these injuries are as follow:—the head of the bone remains in the acetabulum attached by the ligamentum teres. There are, upon parts of the head of the bone, very small white spots like ivory. The cervix is sometimes broken directly transversely, at others with obliquity. The cancellated structure of the broken surface of the head of the bone and of the cervix, is hollowed by the pressure of the neck attached to the trochanter, and consequent absorption; and this surface is sometimes partially coated with a ligamento-cartilaginous deposit. The cancelli are rendered firm and smooth by friction, as we see in other bones which rub upon each other when their articular cartilages are broken off. Portions of bone are formed or broken off, and these are found either attached by means of ligament, or floating loosely in the joint, covered by a ligamentous matter; but these pieces do not act as extraneous bodies, so as to excite inflammation, and thus produce their discharge, any more than those loose portions of bone covered by cartilage, which are found so frequently in the knee, and sometimes in the hip and elbow-joints. With respect to the neck of the bone which remains attached to the trochanter major, the most remarkable circumstance is, that it soon becomes in a great degree absorbed, only a small portion of it remaining; its surface is yellow, and extremely smooth, if the bones have rubbed against each other. Some ossific disposition I have seen manifested around this small remaining part of the neck of the bone, and upon the trochanter major of the thigh-bone below it, in several examples of this fracture. We do not, however, observe the same process of union as in other bones, but a ligamentous instead of an ossific union.

The trochanter is drawn up, more or less, in different accidents; and in those cases in which it is very much elevated, I have known a considerable ossific deposit take place upon the body of the thigh-bone, between the trochanter major and the trochanter minor. When the bone has been macerated, its head is much lighter and more spongy than in the healthy state, excepting on those parts most exposed to friction, where it is rendered smooth by the attrition which gives it a polished surface."

The following are the principles laid down for distinguishing the different species of fracture of the thigh.

"*The fracture of the cervix within the capsule* is known, with very rare exceptions, by the very advanced age of the patient,—by its greater frequency in female than in male subjects,—by the absence of swelling and ecchymosis,—by the elevation and advance of the trochanter,—by the greater mobility of the joint, allowing flexion and extension, although with some pain, and resistance from muscles,—by a crepitus perceptible only on drawing down the limb to the same length with the other, and then rotating it,—by the pain felt at the trochanter minor,—by the small degree of constitutional irritation attending the accident,—by the slight causes which produce it,—and by the little local swelling or change of appearance which ensues.

*Fractures of the cervix into the cancelli of the trochanter* are known by the effusion of blood amidst the muscles,—by great swelling produced, and by ecchymosis, which appears soon after the accident,—by an unnaturally fixed state of the joint, so that flexion and extension cannot be performed,—by excessive pain on the least motion of the hip-joint, and upper part of the thigh-bone,—by a crepitus attending the least motion of the thigh-bone without drawing it down to the length of the other,—and by the inflammation, swelling, and constitutional irritation produced, which are frequently fatal.

*The fracture of the trochanter major* may be easily known by the separation of the bone at the part, so that the finger may be placed between the fractured portions,—by the distinct crepitus felt in putting the fingers on the trochanter when the knee is advanced, by inability of the upper portion of the trochanter to obey the motions of the lower, and of the shaft of the bone,—and when at the lower part of the trochanter, by great overlapping, distension, and exhuberant callus."

We give his opinion upon the proper position of the thigh when fracture takes place below the trochanter. The difficulty in this case arises from the impossibility of preventing the iliacus, internus, and psoas muscles from drawing the upper portion of the bone upwards.

"To prevent this horrid distortion and imperfect union, two circumstances are to be strictly observed; the one is, to elevate the knee very much over the double inclined plane; and, the other, to place the patient in a sitting position, supporting him by pillows during the process of union. The degree of elevation of the body which is required will be about forty-five degrees, but it may be readily ascertained by observing the approximation of the fractured extremities of the bones; and this position is requisite for relaxing the psoas iliacus muscles, and thus preventing the elevation of the upper part of the bone. In no other manner can the great deformity I have described be prevented. When, by this posture, the extremities of the bones are brought into proper apposition, and all projection of its upper portion is removed, either the splints may be applied which are commonly used in fracture of the thigh-bone, or, what is better, a strong leathern belt, lined with some soft material, should, by means of several straps, be buckled around the limb, and be confined by means of a strap around the pelvis.

We shall close our account of this work with a review of the remarks upon compound dislocation of the ankle joint, in which the author has summed up the principles of treatment which should regulate our practice in similar injuries of the other joints.

The first symptom, after the exposure of the joint, is a profuse discharge of the synovia, increasing with the determination to the part which immediately takes place. The inflammation, in about five days, proceeds to suppuration, which, eventually, becomes very abundant. Ulceration of the cartilages succeeds, which sometimes proceeds so far as to lay the foundation for exfoliation of the extremities of the bones. When the cartilages are absorbed, granulations arise from the surface of the bones, and these inosculate, and fill the cavity between the extremities of the bones. Such is the general process. Sometimes, however, adhesive inflammation takes place, and the surfaces of the joints adhere without ulceration.

This inosculation of granulations, or adhesive inflammation, does not generally produce permanent stiffness of the joint, provided passive motion be commenced as soon as the cessation of the pain and inflammation will permit. Even if the structure of the joint is destroyed, a new articulating surface is formed, and the partial stiffness of the ankle which may result, is compensated by an increased mobility of the other joints of the tarsus.

The constitutional disturbance arising from this local inflammation runs very high, and if the local irritation be great, the nervous system is very much affected, and watchfulness, delirium, and subsultus tendinum, and sometimes tetanus, ensue.

Thirty years ago it was the practice to amputate the limb for this accident; but it is now well known that in the greater number of cases, this operation is unnecessary. Every extraneous matter, such as dirt, loose pieces of bone, &c. is to be carefully removed from the wound, and, if necessary for this purpose, the wound in the integuments is to be enlarged.

*Reduction.*—The mode of reducing the bone is, in other respects, similar to that which I have already described, when speaking of the simple dislocation; bending the leg upon the thigh, so as to relax the muscles before the extension is made. When the bone has been reduced, a piece of lint is to be dipped in the patient's blood, and applied wet over the wound, upon which the blood coagulates, and forms the most natural, and, as far as I have seen, the best covering for the wound. A many-tailed bandage is then applied, the portions of which should not be sown together, but passed under the leg, so that any one piece may be removed when it becomes stiff; and by fixing another to its end, the application may always be renewed without any disturbance to the limb: this bandage is to be kept constantly wet with spirits of wine and water. A hollow splint, with a foot-piece at right angles, is to be applied on the outer side of the leg, in the dislocation inwards, and the leg is to rest upon its outer side: but in the dislocation outwards, it is best to keep the limb upon the heel, with a splint and foot-piece both upon the outer and the inner side: and an aperture in the splint opposite to the wound."

Sir Astley is not very favourably disposed to blood-letting in this injury, thinking it important, as a general principle, to husband the strength for the great trial which the powers of life will have to undergo. And therefore, he thinks whatever is to be done, either in the way of bleeding or purging, should be done early after the accident, before adhesive inflammation has taken place. An opinion very different from that entertained by many respectable surgeons, who think that where the inflammatory symptoms run high, the constitution is more impaired by the unrestrained course of inflammatory action, than by the loss of blood. In the greater number of the cases recorded in the present volume, blood-letting was seldom resorted to, to any extent. Irritation should be allayed by the spts. Mindereri and opiates, with occasionally a mild aperient.

"*Secondary treatment.*—If the patient complain of considerable pain in the part, in four or five days, the bandage may be raised to examine the wound; and if there be much inflammation, a corner of the lint should be lifted from the wound, to give vent to any matter which may be formed; but this ought to be done with great circumspection, as there is a danger of disturbing the adhesive process, if that be proceeding without suppuration. By this local treatment, it will every now and then happen that the wound will be closed by adhesion; but if in a few days it be not, and if suppuration take place, the matter should have an opportunity of escaping; and the lint being removed, simple dressing should be applied. After a week or ten days, if there be suppuration, with much surrounding inflammation, poultices should be applied upon the wound, leeching in its neighbourhood, and upon the limb at a distance, and the evaporating lotion should be still employed; but as soon as the inflammation is lessened, the poultices should be discontinued, as they encourage too much secretion, relax the blood-vessels of the part, so as to prevent the restorative process."

The practice has been often pursued of sawing off the end of the tibia before restoring the bone to its position. The following quotation presents a full view of the subject.

"*Sawing off the ends of the bones, &c.*—There is another mode of treatment in these accidents, which consists in sawing off the extremity of the tibia before the bone is returned into its natural situation; and the reasons which may be assigned for pursuing this practice are as follow.

*Difficult reduction.*—First. That there is in some cases much difficulty in the reduction of the tibia, and great violence must be employed to affect it.

*Oblique fracture.*—Secondly. The extremity of the bone is often broken obliquely, so that when reduced it will not remain upon the astralagus, but when the point is removed by the saw, it rests without difficulty upon that bone.

*Spasms.*—Thirdly. The spasmodic contractions of the muscles are much diminished by shortening the bone, as it throws them all into a state of relaxation; whereas, if the bone be reduced by violence when the saw has not been used, the spasm of the limb will be sometimes very violent.

*Local irritation diminished.*—Fourthly. The local irritation is much diminished by the greater ease with which adhesion of the sawn extremity of the bone to the parts to which it is applied; for it is a mistake to suppose that the sawn end of the bone will not adhere; the contrary is seen in amputation in sawing off a bone in exostósis, and in the union by adhesion of compound fractures; and that adhesive matter can be thrown out upon cartilaginous surfaces is known to every person who has dissected a diseased joint; it is thus that the end of the tibia adheres to the surface of the astralagus.

*Suppuration and ulceration lessened.*—Fifthly. When suppuration does occur it is much diminished, and a considerable part of the ulcerative process is prevented by the mechanical removal of the cartilage; for nearly half the articular surface of the joint no longer remains. Cæteris paribus, therefore the case recovers more rapidly.

*Less constitutional irritation.*—Sixthly. The constitutional irritation is very much lessened by the diminution of the suppurative and ulcerative process, and by the ease with which the parts are restored. In the cases which I have had an opportunity of seeing, there was not more irritative fever than in the mildest cases of compound fracture.

*Bone shattered.*—Seventhly. It has been found that in cases in which the extremities of the bones forming the joint have been broken into small pieces, and in which these have been removed by the finger, the patient has suffered less, and has more quickly recovered, than when the bone has been returned whole.

*No case of death.*—Eighthly. I have known no case of death when the extremities of the bones have been sawn off, although I shall have occasion to mention some in which the cases terminated fatally when this was not done.

*Objections; limb shorter.*—The objections which may be made to this mode of treatment, are, that the limb becomes somewhat shorter by the removal of the cartilaginous extremity of the bone; but this I do not think an objection of any considerable weight, if the danger of the case be, as I believe, lessened by it; for the diminished length, which is very slight, is easily supplied by a shoe made a little thicker than usual.

*Anchylosis.*—The other objection is, that the joint becomes necessarily anchylosed. I doubt very much the reality of this objection, as in two instances I have seen the motion of the part remain; but even when the joint becomes anchylosed, a consequence to which it is liable in either mode of treatment, the motion of the tarsal bones becomes so much increased as to compensate for that of the ankle, the patient walks with much less halting than would be anticipated, and has a very useful limb.

*Each mode useful.*—My intention, however, is not to advocate either mode of treatment to the exclusion of the other, but to state the reasons which may be justly assigned for the occasional adoption of either. It is only by a comparison of the different results of varied practice that a safe conclusion can be drawn; and from what I have had an opportunity of observing in my own practice, and of learning from that of my friends, I feel disposed to recommend to those whose minds are not settled upon the subject, not hastily to determine against either treatment in the different cases of this injury, as from each mode, under varied circumstances, a strong and useful limb has been saved without any additional risk to the life of the patient.

*Cases in which the one or the other should be employed.*—If the dislocation can be easily reduced without sawing off the end of the bone; if the bone be not so obliquely broken, but remain firmly placed upon the astralagus when reduced; if the end of the bone be not shattered, for then the small loose pieces of bone should be removed, and the surface of the bone be smoothed by the saw; if the patient be not excessively irritable, so as to occasion the muscles to be thrown into violent spasmodic actions in the attempt at reduction, which leads to subsequent displacement when the limb has been reduced; the bones should be at once returned into their places, and the parts should be united by the adhesive inflammation; but rather than amputate the limb, if the above circumstances were pesent, I should certainly saw off the ends of the bones."

Although in the majority of cases the limb may be saved, yet occasionally amputation will be necessary. With the following abstract of the circumstances under which it will be required, we will close this article.

1. *The advanced age of the patient.*

2. *A very extensive lacerated wound.*

3. *A difficulty in reducing the bones* has been considered a reason for amputation; but as this difficulty may always be obviated by sawing off the extremity of the bone, it should never be considered as a sufficient excuse for destroying the limb.

4. *A comminated fracture of the bones.*

"*Bones shattered.*—If the lower extremity of the tibia be broken into small pieces, the loose portions of bone ought to be removed, and the end of the tibia to be smoothed by a saw; but if, in addition to this comminution, the lower extremity of the tibia be obliquely broken, and a large loose portion of bone be felt with the fingers, then it will be proper to amputate: also, if the astralagus be broken, the portions of this bone should be removed, otherwise they will separate by ulceration, or occasion considerable local irritation."

5. *The dislocation of the tibia at the outer ankle.*

"The dislocation of the tibia at the outer ankle, produces much more injury and danger than that at the inner, and amputation will be more frequently required for it, because both the bones and soft parts suffer more than in the dislocation inwards."

6. *It sometimes happens that when the bone is replaced it will not remain in its situation, and all the symptoms of the injury become renewed.*—This circumstance happens when the tibia, in the dislocation outwards, is obliquely fractured, and as only a small portion of the articulating extremity remains on the dislocated extremity of the tibia, it will not rest on the tibia when it is reduced.

7. *The division of a large blood-vessel* might, with an extensive wound of the integuments, lead to a necessity for amputation. Sir Astley thinks, however, that on account of the free anastomes of the arteries about the ankle joint, there will not be a necessity for amputating, unless the divison of the blood-vessel be accompanied with other untoward symptoms.

8. *Mortification of the foot.—Excessive contusion.—Extensive suppuration.*

9. *Exfoliations of bone*, which from being locked in the surrounding portions of bone, are incapable of being separated, and thus keep a continued state of irritation.

"My friend, Mr. Hamminck, had the kindness to send me a specimen of this kind, which he was obliged to amputate. The loose portion of the bone was seated between the lower extremity of the tibia and fibula, and reached to the ankle-joint; both the bones had been broken and had become reunited, and the uniting medium had inclosed and incarcerated the dead portion of bone. It is probable, from the appearance of the parts, that this portion of bone never would have been able to escape from the place in which it was locked."

10. *Excessive deformity of the foot.*

11. *Tetanus.*—Sir Astley disapproves of the practice of operating for the purpose of relieving the disease, the operation having appeared to precipitate a fatal issue. The practice of amputating upon the appearance of this disease was introduced by Baron Larrey with very high expectations, but the experience of other surgeons has not confirmed his sanguine anticipations.

12. *Corpulent persons*, unless they are in the habit of taking active exercise, suffer more from injuries than others, and therefore are frequently compelled to submit to amputation, on account of injuries which would otherwise be attended with little difficulty.

We regret that we are here under the necessity of suspending our analysis of this valuable work. We do this the more willingly, however, because no abstract of the individual opinions of the author can supply the benefit which the student of surgery will derive from the recorded cases, illustrating every variety of accident, which could with propriety be introduced into a work of this kind. In addition to this, there is appended to the work, a valuable set of plates, which very much enhances its value.

In this country, where every physician is compelled to unite in his own person all the various departments of the profession, this volume should form a part of every medical library. Cases of surgery form a very important point of each individual's practice, and upon the dexterity and success with which he attends to them, will depend much of his reputation. It has been satirically said of our profession, that the grave conceals the errors of the physician; not so of the surgeon, whose patients frequently remain palpable monuments of his ignorance or carelessness.

